# Clinical Evaluation of High-Volume Hemofiltration with Hemoperfusion Followed by Intermittent Hemodialysis in the Treatment of Acute Wasp Stings Complicated by Multiple Organ Dysfunction Syndrome

**DOI:** 10.1371/journal.pone.0132708

**Published:** 2015-07-24

**Authors:** Xiaoyun Si, Jingjing Li, Xiaohong Bi, Lan Wu, Xiaoyan Wu

**Affiliations:** Department of Nephrology, Zhongnan Hospital of Wuhan University, Wuhan 430071, Hubei Province, China; Bambino Gesù Children's Hospital, ITALY

## Abstract

Multiple organ dysfunction syndrome (MODS) is a rare complication of wasp stings. Currently, there is no standardized treatment for MODS secondary to multiple wasp stings, although blood purification techniques are often used. This study aimed to analyze our experiences of using intermittent hemodialysis (IHD) with or without high-volume hemofiltration (HVHF) for treating acute wasp stings complicated by MODS. In this retrospective study, 36 patients with wasp stings complicated by MODS received either IHD combined with hemoperfusion, or HVHF (ultrafiltration flow rate, 70 mL/kg/h) combined with hemoperfusion for 5 days followed by IHD. Clinical symptoms, blood biochemical parameters, duration of mechanical ventilation, use of vasoactive agents, duration of hospital stay and survival rate were recorded, and Acute Physiology and Chronic Health Evaluation II (APACHE II) and multiple organ dysfunction (MOD) scores estimated. Patients treated with HVHF followed by IHD appeared to exhibit a faster recovery than those receiving IHD alone, as evidenced by superior improvements in MOD (4.29±1.08 *vs*. 2.27±1.07) and APACHE II (7.09±2.62 *vs*. 4.20±1.69) scores (*P* < 0.05). Patients treated with HVHF had significantly lower myoglobin, creatine kinase-MB, lactate dehydrogenase, bilirubin and creatinine levels than patients treated with IHD alone. In addition, the durations of hospital stay (13.15±2.77 *vs*. 27.92±3.18 days), vasopressor use (1.76±0.24 *vs*. 3.43 ± 1.01 days), mechanical ventilation (3.02±1.63 *vs*. 5.94 ± 2.11 days) and oliguria (6.57±2.45 *vs*. 15.29 ± 3.51 days) were reduced, and renal function more often recovered (85.1% *vs*. 53.1%), in the HVHF group compared with the IHD group (*P* < 0.05). These results raise the possibility that HVHF plus IHD may be superior to IHD alone for the treatment of acute wasp stings complicated by MODS; additional prospective studies are merited to explore this further.

## Introduction

Reactions to wasp stings can take on a wide variety of forms, ranging from mild and local to systemic and potentially fatal. These reactions are caused by components of the wasp sting, including phospholipase A, hyaluronidase and various polypeptides. These components can induce the release of inflammatory mediators, such tumor necrosis factor, interleukin-1 (IL-1), IL-6 and IL-8. Although severe envenoming is rare, it can lead to multiple organ dysfunction syndrome (MODS). MODS has various clinical manifestations, including intravascular hemolysis, rhabdomyolysis, acute renal failure, hepatic injury, myocardial injury, disseminated intravascular coagulation (DIC), respiratory failure and nervous system damage [1–6]. The majority of patients with MODS, including MODS secondary to insect stings, are treated with blood purification therapy to support renal function and remove toxic compounds [7–10]. However, since the overall incidence of MODS secondary to multiple wasp stings is very low, there have been no definitive studies in humans to establish the optimal treatment regimen. As a result, currently there is no standardized therapy for wasp stings complicated by MODS.

One blood purification approach that has been used in the treatment of MODS is intermittent hemodialysis (IHD), which relies on the passive diffusion of solutes through a semi-permeable membrane. However, although IHD can clear small molecules of masses up to 500 Da, it cannot clear the larger protein components of wasp venom and the associated inflammatory mediators; furthermore, the efficacy of a single treatment is limited [7, 8]. Continuous veno-venous hemofiltration (CVVH) is an alternative technique that provides continuous blood purification based on convection driven by a hydrostatic pressure gradient. This method has theoretical advantages over IHD in that convection is more effective at eliminating middle molecular weight molecules (5–60 kDa) such as inflammatory mediators, and is now considered the therapy of choice in critically ill patients requiring renal replacement [8]. High-volume hemofiltration (HVHF) is a form of CVVH that is generally defined as having an ultrafiltration flow rate > 50 mL/kg/h [8]. HVHF followed by IHD appears to have advantages over IHD alone in the treatment of bee stings, evidenced by earlier declines in bilirubin, alanine aminotransferase, creatine kinase and white blood cell levels [9]. Furthermore, HVHF has been shown to be beneficial for severely ill patients with systemic inflammatory states, and for the treatment of wasp stings [8, 10].

In order to gain better insight into the relative merits of IHD and HVHF in the treatment of wasp stings complicated by MODS, we carried out a retrospective analysis of our clinical experiences of using IHD with or without HVHF.

## Materials and Methods

### Patients

From September 2008 to December 2011, 36 patients with wasp sting poisoning complicated by MODS were admitted to the Department of Nephrology, Zhongnan Hospital (Wuhan, China). Diagnosis was made according to the criteria for MODS [11], including confirmed wasp stings, and the occurrence of dysfunction or failure of 2 or more organs successively or simultaneously 24 h after the wasp stings. An organ dysfunction score ≥ 2 was defined as organ failure. After being informed about the disease condition, treatment regimen, complications and treatment cost, the patients or their family members were allowed to select between HVHF plus IHD or IHD alone. Since not all patients were insured and were required to pay for the treatment received (often in advance of treatment), it is likely that the cost of therapy (with HVHF plus IHD being more expensive than IHD alone) was an important influence on the decision made by each patient.

Twenty-one patients received HVHF for 5 days followed by IHD, and 15 patients received IHD alone. Clinical features and treatment outcomes were retrospectively analyzed. This study was approved by the Ethics Committee of Zhongnan Hospital of Wuhan University. Patient records/information was anonymized and de-identified prior to analysis. As this was a retrospective analysis of anonymized data extracted from clinical records, informed consent from each patient for inclusion in the analysis was deemed not to be required.

### Integrated treatment

After admission to hospital, patients received wound debridement, reduced glutathione and omeprazole (for liver protection), antibiotics (for prevention and control of infections), and anti-allergy medications. An artificial airway was established to assist respiration in patients with coma or respiratory dysfunction, and vasoactive agents were given to patients with circulatory instability.

### Intermittent hemodialysis

**Fig 1:**
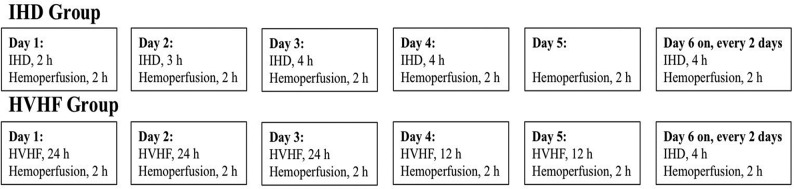
Scheme to illustrate the blood purification treatments used in the two patient groups. Patients in the IHD group underwent IHD in combination with hemoperfusion. Hemoperfusion in the IHD group was carried out for 2 h each day, with a blood flow of 150–200 mL/min. IHD was administered for 2 h on day 1, 3 h on day 2, 4 h on day 3, and then 4 h every other day, with a dialysis liquid flow rate of 500 mL/min, and a blood flow of 200–300 mL/min. Patients in the HVHF group underwent HVHF in combination with hemoperfusion (2 h each day, with a blood flow volume of 150–200 mL/min). During the first 72 h of treatment the hemofiltration solution flow rate was 70 mL/kg/h, blood flow was 200–250 mL/min, and the filter and catheter were changed every 24 h; subsequently, hemofiltration solution was given at 70 mL/kg/h for 12 h each day.

Patients in the IHD group underwent IHD in combination with hemoperfusion (Fig 1) through an emergency indwelling dual-lumen catheter placed in the right femoral vein, using a Dialog+ dialysis machine (Braun Avitum Trading Co. Ltd, Melsungen, Germany) and bicarbonate dialysate. The resin hemoperfusion apparatus (HA330-II; Zhuhai Livzon Diagnostics Inc., Zhuhai, China) was connected to a Fresenius AV600 dialyzer (Fresenius Medical Care, Bad Homburg, Germany; polysulfone membrane, with a membrane area of 1.4 m^2^). Hemoperfusion was carried out for 2 h each day, with a blood flow of 150–200 mL/min. IHD was administered for 2 h on day 1, 3 h on day 2, 4 h on day 3, and then 4 h every other day, with a dialysis liquid flow rate of 500 mL/min, and a blood flow of 200–300 mL/min.

### High-volume hemofiltration

Patients in the HVHF group underwent HVHF in combination with hemoperfusion (Fig 1) through an emergency indwelling dual-lumen catheter, placed in the right femoral vein, using an Aquarius hemodialysis system (Baxter International Inc.; Deerfield, IL, USA) and bicarbonate dialysate. The resin hemoperfusion apparatus (HA330-II; Zhuhai Livzon Diagnostics Inc.) was connected to a Fresenius AV600 hemodialysis system (Fresenius Medical Care) for 2 h each day. The blood flow volume was 150–200 mL/min. Hemofiltration solution (140 mmol/L Na^+^, 4.0 mmol/L K^+^, 0.8–1.0 mmol/L Mg^2+^, 1.5 mmol/L Ca^2+^, 4.5–6.0 mmol/L glucose and 35 mmol/L HCO_3_^-^) was transfused using a pre-dilution method. During the first 72 h of treatment the hemofiltration solution flow rate was 70 mL/kg/h, blood flow was 200–250 mL/min, and the filter and catheter were changed every 24 h (to reduce the risk of thrombosis); subsequently, hemofiltration solution was given at 70 mL/kg/h for 12 h each day. Low molecular weight heparin was administered as an anticoagulant (an initial dose of 2000 U followed by administration at 200 U/h; administration was stopped 1 hour before dialysis), unless bleeding or bleeding tendency was observed. HVHF was performed for 5 days, followed by IHD.

### Clinical characteristics

Clinical features were extracted from patient medical records. The change in the multiple organ dysfunction (MOD) score was selected as the primary outcome measure, with numerous secondary outcome measures also assessed, as follows. The MOD score and Acute Physiology and Chronic Health Evaluation II (APACHE II) score were estimated as described previously [12]. Level of consciousness, blood pressure, respiratory characteristics, urine volume and color, ratio of the partial pressure of oxygen in arterial blood (PaO_2_) to the inspired oxygen fraction (FiO_2_), and the presence or absence of jaundice, gastrointestinal tract symptoms (nausea, vomiting or anorexia) and fever were determined. The mean durations of mechanical ventilation, administration of vasoactive agents and hospitalization, and the survival rate were recorded during hemodialysis. Venous blood was collected from an antecubital vein, with the patient in a resting state, daily for 5 days after dialysis. The platelet count, prothrombin time, hemoglobin level, serum total bilirubin, serum direct bilirubin, serum indirect bilirubin, serum creatinine, blood urea nitrogen, myoglobin, creatine kinase-MB isoenzyme (CK-MB), aspartate aminotransferase (AST), alanine aminotransferase (ALT) and lactate dehydrogenase (LDH) were measured using an Olympus AU5400 fully automatic biochemical analyzer (Olympus Corp., Tokyo, Japan). Hepatic dysfunction was diagnosed when ALT exceeded twice the normal upper limit, or serum total bilirubin exceeded 34.2 μmol/L (> 2.0 mg/dL) [11].

### Statistical analysis

All continuous data are expressed as the mean ± standard deviation (SD), while categorical data are described as numbers and percentages. Normal distribution of data was tested with the Kolmogorov–Smirnov test. Comparison between quantitative data was performed with two-sample *t*-test (or Mann-Whitney U test for non-normally distributed data). Fisher’s exact test was employed for comparisons of categorical data. Multiple comparisons were performed using repeated measures ANOVA. All statistical analyses were carried out using SPSS version 15.0 statistical software (SPSS Inc., Chicago, IL, USA). *P* < 0.05 was considered statistically significant.

## Results

### Patient clinical characteristics

The 36 patients admitted to hospital following wasp stings included 7 females (19.4%) and 29 males (80.6%); their demographic and clinical characteristics are presented in Table 1. Before admission, none of the patients had any previous medical history of heart, liver, kidney, brain, lung or connective tissue disease, diabetes, hypertension, malignant tumors, or use of immunomodulatory drugs (including glucocorticoids). The sting sites were located mainly on the head, face, neck, upper and lower limbs, chest and back. Clinical symptoms included erythema, swelling, burning sensation, tingling and pruritus at the wasp sting sites. The time between receiving stings and medical consultation was 10–96 h (54.5±24.1h). There were no significant differences between patients receiving HVHF plus IHD and those receiving IHD alone in age, gender, organ failure, duration of time from the wasp sting to treatment (2.61 ± 1.02 *vs*. 2.22 ± 1.14 days) or number of sting wounds (16.53 ± 4.77 *vs*. 14.96 ± 5.18) (Table 1; *P* > 0.05).

**Table 1:** Demographic and clinical characteristics of the patients in the HVHF and IHD groups.

	HVHF	IHD	*P*
No. of patients	21	15	
Male/female	17/4	12/3	1.000
Age, years (mean ± SD)	40.3 ± 2.6	41.6 ± 2.2	0.136
Days to receiving treatment, days (mean ± SD)	2.61 ± 1.02	2.22 ± 1.14	0.284
Number of sting wounds, n (mean ± SD)	43.67 ± 10.66	42.40 ± 9.57	0.368
Circulatory failure, n (%)	8 (38.1)	5 (33.3)	1.000
DIC, n (%)	4 (19)	2 (13.3)	1.000
Hemolysis, n (%)	19 (90.4)	13 (86.7)	1.000
Hepatic dysfunction, n (%)	18 (85.7)	13 (86.7)	1.000
Myocardial damage, n (%)	17 (80.9)	12 (80.0)	1.000
Organ dysfunction, n (mean ± SD)	3.48 ± 0.98	3.53 ± 1.12	0.872
Renal failure, n (%)	11 (52.4)	8 (53.3)	1.000
BUN, mmol/L (mean ± SD)	30.48 ± 8.12	31.33 ± 9.52	0.773
Scr, μmol/L (mean ± SD)	314.05 ± 48.73	295.53 ± 39.39	0.233
Respiratory failure, n (%)	7 (33.3)	5 (33.3)	1.000

*Note*: BUN, blood urea nitrogen; DIC, disseminated intravascular coagulation; HVHF, high-volume hemofiltration; IHD, intermittent hemodialysis; SD, standard deviation; Scr, serum creatinine. Circulatory failure, myocardial damage, DIC, organ dysfunction, renal failure and respiratory failure are defined as in references 13 and 14.

At admission, there was acute hemolysis (diagnosed by the observation of fractured or metamorphotic red cells, an increased reticulocyte of 5–20%, and a plasma free hemoglobin level > 40 mg/L) in 32 patients (88.9%), abnormal liver function in 31 (86.1%), myocardial damage in 29 (80.6)%, acute renal failure in 19 (52.8%), and DIC in 6 (16.7%) (Table 1). Thirteen patients (36.1%) received circulatory support and 12 (33.3%) required mechanical ventilation (Table 1).

### Clinical parameters of patients receiving HVHF and IHD

Before treatment, no significant difference was detected between the two groups in all the clinical characteristics monitored (Table 2). On day 5, patients in both groups experienced significant improvements in APACHE II score, PaO_2_/FiO_2_ ratio, mean arterial pressure, coagulation function (reduced prothrombin time and increased platelet count), and liver and kidney functions (increased hemoglobin level; and reduced blood urea nitrogen, AST and LDH) (Table 2; *P* < 0.05). After treatment, APACHE II score (17.07 ± 2.52 *vs*. 14.24 ± 2.10; *P* < 0.01), MOD score (9.53 ± 2.33 *vs*. 8.00 ± 1.70; *P* < 0.05), myoglobin level (683.1 ± 66.4 *vs*. 250.7 ± 41.8 ng/mL; *P* < 0.05), CK-MB level (98.93 ± 29.07 *vs*. 50.10 ± 18.97 U/L; *P* < 0.05) and LDH level (1486.2 ± 36.1 *vs*. 288.7 ± 16.2 U/L; *P* < 0.05) were significantly lower in patients in the HVHF group compared with those in the IHD group (Table 2). In addition, the total, direct and indirect bilirubin and serum creatinine levels improved significantly in the HVHF group, but not in the IHD group (Table 2; *P* < 0.05). Hemodialysis was ceased in patients without renal failure, but was continued in patients with renal failure until recovery of renal function (defined as the polyuric stage, with urine production > 3 L/d).

**Table 2:** Clinical parameters of the patients in the HVHF and IHD groups before and after treatment.

Parameter	Before treatment		*P* value	After treatment		*P* value	Change after treatment		*P* value
	HVHF group	IHD group		HVHF group	IHD group		HVHF group	IHD group	
APACHE II score	21.33 ± 3.14	21.27 ± 3.62	0.953	14.24 ± 2.10**	17.07 ± 2.52**	0.002	7.09 ± 2.62	4.20 ± 1.69	0.0003
MOD score	12.29 ± 2.51	11.80 ± 2.37	0.562	8.00 ± 1.70**	9.53 ± 2.33	0.047	4.29 ± 1.08	2.27 ± 1.07	< 0.0001
PaO_2_/FiO_2_ (mmHg)	286.55 ± 120.10	291.28 ± 125.59	0.910	407.97 ± 75.48**	390.76 ± 120.81*	0.634			
MAP (mmHg)	60.10 ± 5.22	59.07 ± 5.19	0.563	77.05 ± 10.42**	75.33 ± 13.82**	0.700			
PT (s)	17.33 ± 5.31	17.47 ± 4.42	0.937	12.38 ± 4.80**	12.87 ± 5.38*	0.795			
PLT (×10^9^/L)	62.24 ± 14.22	63.67 ± 18.06	0.792	158.57 ± 15.16**	153.33 ± 17.03**	0.386			
HB (g/dL)	7.395 ± 1.087	7.373 ± 1.216	0.955	9.029 ± 1.492**	8.333 ± 1.115*	0.181			
Myoglobin (ng/mL)	1254.9 ± 186.2	1177.3 ± 162.5	0.203	250.7 ± 41.8**	683.1 ± 66.4	< 0.001			
CK-MB (U/L)	127.43 ± 25.43	128.87 ± 23.57	0.864	50.10 ± 18.97**	98.93 ± 29.07	< 0.001			
AST (U/L)	861.29 ± 60.40	884.33 ± 53.67	0.246	52.62 ± 10.65**	224.73 ± 15.11*	< 0.001			
LDH (U/L)	6011.7 ± 139.8	5967.2 ± 154.8	0.375	288.7 ± 16.2**	1486.2 ± 36.1*	< 0.001			
TBI (μmol/L)	78.95 ± 19.90	88.13 ± 19.06	0.174	37.67 ± 11.53*	66.13 ± 16.08	< 0.001			
DBI (μmol/L)	25.00 ± 12.05	19.53 ± 10.09	0.161	11.95 ± 3.46*	19.47 ± 6.92	< 0.001			
IBI (μmol/L)	53.95 ± 18.42	64.73 ± 17.04	0.083	25.95 ± 9.48*	46.67 ± 11.58	< 0.001			
BUN (mmol/L)	30.48 ± 8.12	31.33 ± 9.52	0.773	12.29 ± 4.62**	14.20 ± 3.39**	0.228			
Scr (μmol/L)	314.05 ± 48.73	295.53 ± 39.39	0.233	189.00 ± 29.85*	274.53 ± 19.45	< 0.001			

*Note*: Data are presented as the mean ± standard deviation.

* *P* < 0.05

** *P* < 0.01 *vs*. that before treatment.

APACHE score, Acute Physiology and Chronic Health Evaluation II; AST, aspartate aminotransferase level; BUN, blood urea nitrogen level; CK-MB, creatine kinase-MB level; DBI, direct bilirubin index; HB, hemoglobin level; HVHF, high-volume hemofiltration; IBI, indirect bilirubin index; IHD, intermittent hemodialysis; LDH, lactate dehydrogenase level; MAP, mean arterial pressure; MOD score, multiple organ dysfunction score; PLT, platelet count; PT, prothrombin time; Scr, serum creatinine level; TBI, total bilirubin index.

PaO_2_/FiO_2_ ratio and mean arterial pressure were significantly improved 1 day after the initiation of HVHF (Table 3; *P* < 0.05). The levels of myoglobin, CK-MB, LDH and AST were significantly reduced after 1 day of HVHF treatment, while the bilirubin level, renal function, APACHE II score and MOD score were significantly improved 3 days after HVHF treatment (Table 3; *P* < 0.05).

**Table 3:** Comparisons of biochemical parameters before and after HVHF.

Parameter	Day 0	Day 1	Day 2	Day 3	Day 4	Day 5	*P* value^#^
APACHE II score	21.33 ± 3.14	20.14 ± 4.10	19.38 ± 3.80	16.05 ± 3.83*	15.43 ± 4.03*	14.24 ± 2.10*	< 0.001
MOD score	12.29 ± 2.51	11.90 ± 2.02	11.24 ± 1.95	8.95 ± 1.77*	8.86 ± 2.06*	8.00 ± 1.70*	< 0.001
AST (U/L)	861.2 ± 60.4	535.7 ± 58.3*	288.4 ± 55.9*	174.3 ± 61.5*	93.5 ± 45.8*	52.6 ± 10.7*	< 0.001
CK-MB (U/L)	127.43 ± 25.43	78.33 ± 13.72*	65.57 ± 12.57*	56.33 ± 11.25*	58.10 ± 10.37*	50.10 ± 18.97*	< 0.001
Myoglobin (ng/mL)	1254.9 ± 186.2	830.7 ± 52.9*	458.2 ± 48.4*	329.3 ± 45.4*	301.7 ± 49.6*	250.7 ± 41.8*	< 0.001
LDH (U/L)	6011.7 ± 139.8	3176.5 ± 121.2*	2252.3 ± 185.3*	1037.2 ± 128.7*	591.6 ± 86.4*	288.7 ± 16.2*	< 0.001
TBI (μmol/L)	78.95 ± 19.90	66.33 ± 12.72*	58.90 ± 11.64	48.62 ± 13.15*	42.76 ± 9.89*	37.67 ± 11.53*	< 0.001
DBI (μmol/L)	25.00 ± 12.05	24.19 ± 11.03	24.14 ± 12.46	20.86 ± 11.31	15.38 ± 8.73*	11.95 ± 3.46*	< 0.001
IBI (μmol/L)	53.95 ± 18.42	43.67 ± 16.23	34.29 ± 15.93*	29.00 ± 12.18*	27.00 ± 9.10*	25.95 ± 9.48*	< 0.001
MAP (mmHg)	60.10 ± 5.22	73.05 ± 9.57*	74.19 ± 8.47*	76.81 ± 8.88*	75.38 ± 8.91*	77.05 ± 10.42*	< 0.001
PaO_2_/FiO_2_ (mmHg)	286.55 ± 120.10	368.93 ± 103.64*	391.47 ± 102.60*	387.54 ± 106.70*	410.26 ± 102.37*	407.97 ± 75.48*	0.002
BUN (mmol/L)	30.48 ± 8.12	27.43 ± 8.82	19.62 ± 9.02*	15.90 ± 8.88*	14.95 ± 9.74*	12.29 ± 4.62*	< 0.001
Scr (μmol/L)	314.05 ± 48.73	285.71 ± 42.14	234.67 ± 33.81*	206.38 ± 30.76*	208.67 ± 28.43*	189.00 ± 29.85*	< 0.001

*Note*: Data are shown as the mean ± standard deviation.

* *P* < 0.05 *vs*. the value on day 0 post-treatment.

#: Multiple comparisons were performed using repeated measures ANOVA.

APACHE score, Acute Physiology and Chronic Health Evaluation II; MOD score, multiple organ dysfunction score; AST, aspartate aminotransferase level; CK-MB, creatine kinase-MB level; LDH, lactate dehydrogenase level; TBI, total bilirubin index; DBI, direct bilirubin index; IBI, indirect bilirubin index; MAP, mean arterial pressure; PaO_2_, partial pressure of oxygen in arterial blood; BUN, blood urea nitrogen level; Scr, serum creatinine level; HVHF, high-volume hemofiltration; IHD, intermittent hemodialysis.

### Clinical outcomes of patients receiving HVHF and IHD

Discharge from the hospital was defined as the observation endpoint. The duration of hospitalization, vasopressor agent use, mechanical ventilation (where required) and oliguria, and the incidence of complications (including cardiovascular and respiratory complications and abnormalities of coagulation) were significantly lower in the HVHF group than in the IHD group (*P* < 0.05). The mean duration of mechanical ventilation was 3.02 ± 1.63 days for the 7 patients in the HVHF group that required it, compared with 5.94 ± 2.11 days for the 5 patients in the IHD group that required it (Table 4). Fewer patients in the HVHF group required additional blood purification following discharge, suggesting a better recovery of renal function (Table 4; *P* < 0.05). Although 2 patients in the IHD group showed increased urine production, they did not proceed to the polyuric stage, and still had elevated serum creatinine levels. At day 45, these 2 patients were transferred to a local county hospital, where they received continuous IHD treatment until day 56 and 65, respectively.

**Table 4:** Comparisons of clinical outcomes at hospital discharge between patients in the HVHF and IHD groups.

	HVHF	IHD	*P*
Cardiovascular complications^#^, n (%)	2 (9.5)**	11 (73.3)	< 0.0001
Circulatory support^$^, days (mean ± SD)	1.76 ± 0.24**	3.43 ± 1.01	< 0.0001
Blood clotting^##^, n (%)	4 (19)*	9 (60)	0.017
Death, n (%)	3 (14.3)	3 (20.0)	0.677
Hospital stay, days (mean ± SD)	13.15 ± 2.77**	27.92 ± 3.18	< 0.0001
Mechanical ventilation, days (mean ± SD)	3.02 ± 1.63 (7 patients)*	5.94 ± 2.11 (5 patients)	0.0359
Oliguria, days (mean ± SD)	6.57 ± 2.45**	15.29 ± 3.51	< 0.0001
Renal function recovery so that purification is no longer required, n (%)	18 (85.1)*	8 (53.1)	0.039

Note

* *P* < 0.05

** *P* < 0.01 *vs*. the IHD group.

^#^ Cardiovascular complications included palpitations, self-reported chest congestion feeling without abnormity detected with electrocardiogram, arrhythmia and shock.

^##^ Blood clotting included vascular coagulation and elevated PPT and DIC.

$ In occasions when blood pressure is lower than the 90/60mmHg, Noradrenaline was given at 10-12ug/min. After blood pressure turned normal (higher than 90/60mmHg), dose was gradually reduced to 2-4ug/min then cease usage.

There were no significant differences in mortality between the two groups (Table 4). Three deaths were observed in the HVHF group, including 2 due to DIC and 1 due to gastrointestinal tract hemorrhage; and 3 deaths occurred in the IHD group, including 2 due to DIC and 1 due to respiratory failure. One of the patients in the HVHF group that died had received mechanical ventilation for 0.5 d (12 h), while 1 of the patients in the IHD group that died had received mechanical ventilation for 2.15 d (51.6 h).

## Discussion

We retrospectively reviewed the medical records of 36 patients with wasp stings complicated by MODS at admission. Our main findings were that patients treated with HVHF followed by IHD appeared to exhibit a faster recovery than those receiving IHD alone, as evidenced by improvements in MOD and APACHE II scores. In addition, patients treated with HVHF had significantly lower myoglobin, CK-MB, LDH, bilirubin and creatinine levels, and shorter durations of hospitalization, oliguria and vasopressor use. Although limited somewhat by the small number of patients available for study and the retrospective nature of the analysis, our data nonetheless raise the possibility that HVHF may have advantages over IHD alone for the treatment of multiple wasp stings complicated by MODS.

Previous studies have observed that patients admitted to hospital for multiple wasp stings present with various organ dysfunctions, in particular acute kidney injury, rhabdomyolysis, hemolysis, hepatic dysfunction and coagulopathy [7, 13, 14]. Clinical manifestations of MODS in our patients were similar to those reported by others [7, 13, 14], and included acute hemolysis, renal, hepatic and/or respiratory failure, myocardial damage and DIC. One-third of our patients received circulatory support and/or required mechanical ventilation. More than half the patients in our study presented with acute renal failure, in agreement with the findings of other investigators [5, 7]. Acute tubular necrosis is the most common histological finding on biopsy [13], and has been suggested to be secondary to the hemolysis and rhabdomyolysis induced by venom toxicity [7, 13, 14]. Thus, the removal of toxins and restoration of normal renal function may be considered important therapeutic goals in the management of patients with MODS induced by multiple wasp stings.

Blood purification is usually required for patients with multiple insect stings complicated by MODS [7,9,10]. Patients receiving continuous renal replacement therapy at an early stage exhibit improved renal recovery and a reduced requirement for chronic dialysis than those undergoing IHD or intermittent renal replacement therapy [15]. Whereas IHD can only clear small molecules from the blood using passive diffusion, HVHF can also remove middle molecular weight solutes (5–60 kDa) through the displacement of a high-concentration hemofiltration solution, and hence is more effective at clearing plasma cytokines such as TNF-α, IL-1, IL-4, IL-6 and IL-10 [13]. HVHF has also been suggested to enhance lymphatic flow, facilitating the clearance of inflammatory mediators from tissues [8]. These advantages of HVHF would theoretically accelerate the clearance of toxins and inflammatory mediators from the blood and tissues, as well as correct electrolyte and acid-base disturbances, thereby facilitating recovery of organ function [8]. We observed that improvements in the MOD and APACHE II scores and myoglobin, CK-MB, LDH, bilirubin and creatinine levels appeared to occur more rapidly in patients treated with HVHF than in those receiving only IHD, suggesting that HVHF may have advantages over IHD. In line with our findings, others have reported that CVVH is superior to IHD in terms of the time required for recovery of renal function [7]. Nonetheless, two recent meta-analyses have not found benefits of HVHF over standard volume hemofiltration in critically ill patients [16] or patients with septic acute kidney injury [17], in terms of 28-day mortality or other outcome measures. Thus, additional studies are merited to further establish whether HVHF has advantages over IHD in the setting of multiple wasp stings complicated by MODS.

All patients in this study were judged clinically to require administration of fluids. Victims of wasp stings usually present with hypovolemia due to hyperpyrexia, vomiting and hemolysis, and thus require volume expansion [14]. Since hypovolemia and acidic urine are factors known to aggravate acute kidney injury induced by myoglobinuria [15], adequate fluid supplementation would also help limit the nephrotoxic effects of rhabdomyolysis. Nonetheless, it is important to avoid over-hydration, as this may result in pulmonary and myocardial injury. Wasp venom and its metabolites can directly damage pulmonary capillary endothelial cells and alveolar epithelial cells, leading to pulmonary edema [18] that can be aggravated by infusion of large quantities of fluid [19]. Myocardial injury can cause excessive aldosterone and antidiuretic hormone secretion, elevating extracellular fluid volume and cardiac load, and thereby inducing heart failure [20]. Thus, it is essential that appropriate rates and volumes of fluid administration are used. In our study, patients treated with HVHF maintained a stable blood pressure, and required shortened durations of circulatory and respiratory support.

IHD has a short duration and involves a large blood volume, which potentially can result in unstable hemodynamics. HVHF avoids such hemodynamic changes, and is safe and effective in patients with hypotension. HVHF also requires less vasopressor use than IHD, and tends to result in higher urine output [21]. In our study, a gradual elevation in mean arterial pressure was observed in the HVHF group, and the requirement for vasopressor drugs was reduced and shortened compared with the IHD group. A reduced incidence of cardiac dysfunction (e.g. palpitations, chest tightness and arrhythmia) was also observed, suggesting that HVHF may have advantages over IHD in terms of stabilizing hemodynamics. An additional advantage of HVHF is that transfusion of a large volume of hemofiltration solution can rapidly and effectively relieve hyperpyrexia, reduce basal metabolic rate, and decrease oxygen consumption [22–24].

Inflammatory mediator removal is thought to alleviate pulmonary interstitial edema, improve intra-alveolar oxygen diffusion and microcirculatory disorders, and increase tissue oxygen utilization. HVHF has been shown to reduce the plasma concentration of various inflammatory cytokines [25]. In this study, the 7 patients requiring ventilation exhibited a reduced alveolar-arterial oxygen partial pressure difference and an improvement in the PaO_2_/FiO_2_ ratio after the first cycle of HVHF. In addition, our findings seemed to indicate that patients in the HVHF group required less mechanical ventilation than those in the IHD group.

Pachyemia (thickening of the blood) and vascular endothelial injury following wasp stings can activate the intrinsic coagulation pathway to induce DIC. In our study, 6 patients developed hemolysis during their first blood collection, 13 required renewal of the hemodialysis catheter during treatment due to blood coagulation, and 6 developed DIC, 4 of whom died. Under appropriate conditions, the transfusion of a large volume of hemofiltration solution by HVHF can reduce blood viscosity, remove components that cause endothelial injury, improve coagulation function and increase platelet count [26, 27].

This study is not without its limitations. First, this was a retrospective observational study, and thus the influence of extraneous factors on our findings cannot be excluded. Second, this was a single-center study, and thus further research is required to determine the generalizability of our findings. Third, no adjustment was made for possible confounding factors, such as the APACHE II and MOD scores, although baseline characteristics were similar between the two groups. Fourth, only a small number of patients were included, due to the rare nature of the disease, and there was a slight imbalance in the number of patients between groups. Fifth, despite apparent differences between the treatment groups in various measures of morbidity, the mortality rate did not differ significantly. Nonetheless, it cannot be excluded that our study was underpowered (due to the small sample size available) to detect small differences in mortality between the HVHF (14.3%) and IHD (20.0%) groups. Sixth, treatment fees were paid by the patients, most of whom were farmers or unemployed, and many of whom were not insured and hence were required to pay for the treatment themselves. As a result, the choice of treatment was determined mainly by its cost (with HVHF plus IHD being more expensive than IHD alone), and this may have introduced bias. Seventh, more precise assessments of renal function recovery, such as measurements of dialysis-free days, were not used. Appropriately designed, large-scale, multi-center, randomized controlled trials are merited to assess the validity of our results.

## Conclusions

Combining HVHF with IHD may be superior to IHD alone for treating MODS secondary to multiple wasp stings, possibly due to improved clearance of toxins and inflammatory mediators, more stable hemodynamics, and more precise volume control. Further large-scale, randomized, controlled, prospective studies are merited to confirm our results.
